# Astrocytes in the Optic Nerve Are Heterogeneous in Their Reactivity to Glaucomatous Injury

**DOI:** 10.3390/cells12172131

**Published:** 2023-08-23

**Authors:** Ying Zhu, Rui Wang, Anthony C. Pappas, Philip Seifert, Andrej Savol, Ruslan I. Sadreyev, Daniel Sun, Tatjana C. Jakobs

**Affiliations:** 1 Department of Ophthalmology, Massachusetts Eye and Ear Infirmary/Schepens Eye Research Institute, Harvard Medical School, 20 Staniford Street, Boston, MA 02114, USA; 2 Department of Ophthalmology, Stanford University, 1651 Page Mill Road, Palo Alto, CA 94304, USA; 3 Department of Ophthalmology, The First Affiliated Hospital of Northwest University, Xi’an 710002, China; 4 Department of Molecular Biology, Massachusetts General Hospital, Boston, MA 02114, USA; 5 Department of Pathology, Massachusetts General Hospital and Harvard Medical School, 185 Cambridge St., Boston, MA 02114, USA

**Keywords:** glaucoma, astrocytes, optic nerve head, RNA-sequencing, phagocytosis, mitophagy

## Abstract

The optic nerve head is thought to be the site of initial injury to retinal ganglion cell injury in glaucoma. In the initial segment of the optic nerve directly behind the globe, the ganglion cell axons are unmyelinated and come into direct contact to astrocytes, suggesting that astrocytes may play a role in the pathology of glaucoma. As in other parts of the CNS, optic nerve head astrocytes respond to injury by characteristic changes in cell morphology and gene expression profile. Using RNA-sequencing of glaucomatous optic nerve heads, single-cell PCR, and an in-vivo assay, we demonstrate that an up-regulation of astrocytic phagocytosis is an early event after the onset of increased intraocular pressure. We also show that astrocytes in the glial lamina of the optic nerve are apparently functionally heterogeneous. At any time, even in naïve nerves, some of the cells show signs of reactivity—process hypertrophy, high phagocytic activity, and expression of genetic markers of reactivity whereas neighboring cells apparently are inactive. A period of increased intraocular pressure moves more astrocytes towards the reactive phenotype; however, some cells remain unreactive even in glaucomatous nerves.

## 1. Introduction

Glaucoma leads to progressive degeneration and death of retinal ganglion cells and their axons [1,2]. The main risk factors are elevated intraocular pressure (IOP), genetics and a positive family history, and advancing age [3,4,5]. In spite of many recent advances, it is not completely understood why ganglion cells degenerate in glaucoma.

Retinal ganglion cell axons are unmyelinated in the retina and optic nerve head (ONH) until they have cleared the laminar region, which is made up exclusively of astrocytes in mice (“glial lamina”, see Supplementary Figure S1 for a schematic) and of astrocytes supported by a collagenous lamina cribrosa in larger species [6,7,8]. Due to mechanical stress by elevated IOP, the lack of myelin, and possibly vascular factors, axons may be particularly vulnerable in the nerve head region [9,10]. Several studies have identified morphological and gene expression changes in the glial cells of the ONH early in the course of the disease before the first signs of degeneration of ganglion cells are visible in the retina [11,12,13,14,15,16,17,18,19]. Given the close proximity of astrocytes to the unmyelinated axons and reactive changes observed in those astrocytes, they have been implicated in the progression of glaucoma [20,21]. All types of injury to the CNS induce a reactive response in astrocytes and reactive astrocytes can exert many functions, both protective and detrimental to surrounding neurons [22,23,24,25]. Recent studies identified gene expression profiles of astrocytes that assumed a neurotoxic (or type A1) state in neuroinflammation, or a neuroprotective (type A2) state that dominates after ischemic injury [26,27]. However, this classification should not be interpreted as denoting fixed cell types but rather as patterns of astrocyte reactivity in response to signals from their environment [28]. Whether astrocytes take a more neuroprotective or neurotoxic route may depend not only on the nature of the injury., but also on the spatial or temporal distance from the point of injury [29,30,31].

There is evidence that at least in the early stages of glaucoma, the protective role dominates. In the optic nerve (as in other tissues, such as the spinal cord [32,33]), general inhibition of astrocyte reactivity leads to a worse visual outcome and increased ganglion cell death following eye injury [34]. However, it is not yet clear in the context of glaucoma which of the many functions of astrocytes are beneficial for ganglion cell and axon preservation. Optic nerve head astrocytes respond to elevated intraocular pressure with characteristic morphological changes. The processes of astrocytes in uninjured nerves are oriented perpendicularly to the axis of the optic nerve and form glial tubes through which the ganglion cell axons pass as bundles [8]. After elevation of IOP, some astrocyte processes sprout longitudinal processes that often invade the axon bundles and contain degraded mitochondria [14,18]. This behavior is apparently typical of glaucomatous optic nerves; however, in many other ways—axonal swelling, accumulation of abnormal mitochondria, and thickening of blood vessel walls—morphological changes in glaucomatous nerves resemble those that occur during normal aging [35,36]. In the present study, we asked three questions. First, what are the differences between glaucomatous and aged nerves on the level of gene expression? Second, what cellular functions of astrocytes are activated in the optic nerve head after elevation of IOP? And third, do optic nerve head astrocytes behave as a single group, or do individual cells differ in their reactions to elevated IOP? We used a combined approach of RNA sequencing, single-cell quantitative PCR, and morphological analysis of single astrocytes in situ to demonstrate that sprouting of new processes and an up-regulation of phagocytosis are characteristic of glaucomatous, but not aged astrocytes. Furthermore, even though all astrocytes in the optic nerve head apparently belong to a single morphological type [8], their reactive profiles are very heterogeneous. 

## 2. Materials and Methods

### 2.1. Animal Husbandry

All animal studies were carried out in accordance with the Association for Research in Vision and Ophthalmology (ARVO) guidelines for the Use of Animals in Ophthalmic and Vision Research and approved by the Institutional Animal Care and Use Committee of the Schepens Eye Research Institute. Male and female C57bl/6 mice were obtained from the Jackson Laboratory (stock number 000664). C57bl/6 mice for RNA-sequencing were 13 and 40 weeks (10 months) old. The mouse line B6.*hGFAPpr*::EGFP was bred in our colony. The original strain *hGFAPpr*::EGFP was kindly provided by Helmut Kettenmann (Max Dellbruck Center, Berlin, Germany). This strain expresses EGFP stochastically in individual astrocytes throughout the brain and optic nerve [8,37,38]. Since the strain was originally on the FVB/N background and carried the rd1 mutation, we backcrossed it with C57bl/6 mice for >10 generations. All animals were group housed under 12 h dark and light cycles and received water and food at libitum.

### 2.2. Microbead Injection and Tissue Preparation

The microbead occlusion model was used to induce elevated IOP [39,40]. Before microbead injection, the baseline IOP was measured with a TonoLab rebound tonometer (ICare, Espoo, Finland) under 1.5% isoflurane anesthesia delivered via a nose cone. Polystyrene microbeads (15 µm diameter, Invitrogen, Carlsbad, CA, USA) were injected into the anterior chamber of the right eye at a concentration of 1.6 × 10^6^ beads in 1 µL sterile PBS using a beveled glass needle (100 µm tip size) attached to a Hamilton syringe. Control groups received an injection of sterile saline solution. All mice were followed by IOP measurements twice weekly for one month. All IOP measurements were taken at the same time of day (mid-morning) to minimize circadian variation. Cumulative IOP (cIOP) was calculated as the area under the curve for IOP over time (in mmHgDays). At the end of the experiment, mice were euthanized by CO_2_ inhalation, and retinas and optic nerves were dissected. The unmyelinated segments of the optic nerves were identified under a dissecting microscope and stored in an RNAlater tissue storage agent (Invitrogen). Retinas were whole-mounted on nitrocellulose filters, fixed in 4% (*w*/*v*) paraformaldehyde in phosphate-buffered saline (PBS), and stained with a mouse monoclonal antibody against Brn3a (Millipore Sigma, MAB1585, Burlington, MA, USA), a marker for retinal ganglion cells, followed by a fluorescent secondary antibody (donkey anti-mouse IgG, catalog no. 715-586-150, Jackson Immunoresearch, West Grove, PA, USA). For ganglion cell counting, 8 image stacks (2 from each quadrant of the retina) were taken through the ganglion cell layer of each retina at a position equidistant to the border of the retina and optic nerve head. Image stacks were imported into ImageJ and automatically counted by a user-written routine. Ganglion cell densities were averaged for each retina and ganglion cell nuclei per mm^2^ were calculated.

### 2.3. RNA Extraction and RNA-Seq

Unmyelinated portions of 4 right optic nerves (from 4 individual mice) were pooled for each RNA extraction. In all cases, 2 of the nerves were from male, and 2 from female mice. Microbead-injected and saline-injected control nerves were processed in parallel. Total RNA was extracted using RNeasy micro columns (Qiagen, Hilden, Germany) and quantified using a Bio Analyzer 2100 (Agilent, Santa Clara, CA, USA). Only preparations with RNA integrity number > 9 were used for RNA-sequencing (details are summarized in Supplementary Table S1). Four biological replicates were used for each group. RNA-sequencing was carried out in the MGH Next Generation core facility (Department of Molecular Biology at MGH). RNA-seq libraries were constructed using the Clontech SMARTer Ultra Low Input RNA Kit for Sequencing v3 (Takara, Kusatsu, Japan) and sequenced on an Illumina HiSeq2500 instrument, resulting in approximately 30 million reads per sample on average. Transcriptome mapping to full genome sequences for *Mus musculus* (UCSC version mm10) was performed with STAR version 2.3.0 [41]. Read counts for individual genes were produced using FeatureCounts [42]. Differential expression analysis was performed using the DESeq2 R package after normalizing read counts and excluding those genes with normalized read counts equal to 0 across all replicates [43]. Differentially expressed genes were defined based on the criteria of a *p*-value < 0.01 (unpaired *t*-tests) and false discovery rate (FDR) < 0.05. Ingenuity Pathway Analysis (Qiagen) was used for pathway analysis.

### 2.4. Dye Injection into the Superior Colliculus

A cohort of 10 mice were injected with microbeads as described above. A control group of 10 mice received saline injections. On day 13 (i.e., four days before the end of the experiment), MitoTracker^TM^ Red FM (Invitrogen, M22425) was injected into the left superior colliculus of each mouse. This time point was chosen because we wanted to maximize the amount of dye in transit for imaging the optic nerve [44,45]. Mice were anesthetized with ketamine and xylazine, and a small hole was drilled in the skull overlying the superior colliculus. About 0.5 μL of a 200 nM solution of MitoTtracker^TM^ Red FM was injected slowly into the tissue with a glass microneedle attached to a Hamilton syringe [46]. After the injection, the hole was filled with Gelfoam (Pfizer) and the skin was closed with a surgical staple.

### 2.5. Tissue Preparation and Confocal Microscopy

After euthanasia with CO_2_ followed by cervical dislocation, the cranium was opened and the bones and brain were removed, exposing the optic nerves including the optic chiasm. The whole skull base was then fixed in 4% paraformaldehyde for one hour. Then the optic nerves were dissected free from the surrounding tissue, postfixed with 4% paraformaldehyde, and embedded in 6% *w*/*v* agarose in PBS. Serial thick (200 μm) sections of the unmyelinated region of the optic nerve were cut on a Leica VT100S vibratome (Leica Microsystems, Buffalo Grove, IL, USA), mounted in Vectashield (Vector Laboratories, Burlingame, CA, USA), and used immediately for imaging on a Leica SP8 confocal microscope. Image stacks were taken through each optic nerve section using a 63× glycerol immersion objective (numerical aperture 1.4). Image stacks that were to be used for quantification were taken without additional optical zoom at the same laser settings, scan speeds (400 Hz), and pixel size (1024 × 1024 pixels, 0.4 μm/pixel). The z-step size was matched to the pixel size in the x and y planes (0.4 μm/pixel). Some cells were imaged at higher magnification to show fine morphological detail. These image stacks were not used for quantification.

For immunohistochemistry, optic nerves were cryoprotected overnight with 30% *w*/*v* sucrose in PBS, embedded in OCT compound, and sectioned at 16 µm. Sections were incubated on the slides in primary antibodies (rat monoclonal anti-galectin 3, Cedar Lane CL8942AP; chicken polyclonal anti-GFAP, Abcam, AB4674; and rabbit polyclonal anti-Iba1, Fujifilm-Wako 019-19741). Secondary antibodies (donkey anti-rat, chicken, or rabbit conjugated to FITC or TRITC were obtained from Jackson Immunoresearch).

### 2.6. Transmission Electron Microscopy (TEM)

The tissue preparation for TEM is described in detail in an earlier publication from our laboratory [35]. Sections from young (3 month), old (10 month), and microbead-injected (1 month after injection) nerves were used to image optic nerve astrocytes. Astrocyte process thickness was measured on TEM images at medium (2900×) magnification. Astrocyte process thickness was measured in young, old, and microbead-injected nerves using ImageJ.

### 2.7. Image Analysis

Confocal image stacks (8 bit per color channel) were imported into Matlab (The Mathworks, Natick, MA, USA) and split into the red channel (containing the signal from the MitoTracker^TM^ Red FM) and green channel (containing the signal from EGFP-labeled astrocytes). The red channel was converted to black and white, median filtered with a kernel size of 3 × 3 pixels, and mitochondria were identified using the bwlabeln function in Matlab. This function returns matrices of all connected components (i.e., mitochondria). As most of these mitochondria were not inside an EGFP-labeled astrocyte, in the second step the identified mitochondria were compared with the pixel values in the green channel. All labeled mitochondria that were completely surrounded by green signal from astrocytes (defined as a pixel intensity value of >100 in the green channel) were identified by a user-written algorithm. In addition, total astrocyte volume and total mitochondria volume were measured by the same algorithm. The processed image stacks were reconstructed using the Amira software package (Visage Imaging, San Diego, CA, USA). The total number of astrocytes and number of MitoTracker^TM^ Red FM-containing astrocytes in each stack was determined with ImageJ by an observer ignorant of the IOP history of the eye.

### 2.8. Single-Cell RT-PCR

Naïve 13- and 40-week-old C57bl/6 mice (3 animals per group) were used for the age comparison of astrocyte gene expression. In addition, microbead-injected mice and saline-injected control mice were used (4 animals per group). Unfixed optic nerve heads were dissected free of the surrounding tissue and separated from the myelinated region of the nerve under microscopic control. The tissue was digested with papain and dissociated into single cells as described before [47,48]. Individual astrocytes were collected into thin wall PCR tubes containing the buffer for reverse transcriptase and PCR (Access PCR System, Promega, Madison, WI, USA), primers for phagocytic markers, reactive astrocytes of neuroprotective and neurotoxic types [49], positive and negative controls. All primer sequences were chosen from the Harvard Primer Bank [50] or designed and tested in-house. Primer sequences are given in Supplementary Table S2. Reverse transcription and first-round amplification were carried out for all primers simultaneously using the Access PCR system with 17 cycles. Second-round PCR or qPCR was carried out for all genes separately on a Lightcycler 480 Instrument (Roche Applied Science, Penzberg, Germany). Gapdh and Oaz1 were used as internal controls and cells that did not express detectable levels (defined as a Ct value of >32) of both genes were not analyzed.

### 2.9. Statistical Analysis

All statistical analysis was carried out in Matlab (The Math Works, Natick, MA, USA) or R (The R foundation). For microbead injection experiments, the sample size needed to detect a 15% loss of ganglion cells at α = 0.05 with a power of 0.8 is *n* = 6; however, for RNA-sequencing larger group sizes (*n* = 16) were used because a single optic nerve does not yield sufficient RNA for one biological replicate. For the experiment using MitoTracker^TM^ Red FM, group sizes of *n* = 10 were used. Single-cell PCR has a high variability. We calculated that *n* = 12 cells per condition would be sufficient to detect a 2-fold change in expression levels with α = 0.05 at 0.8. Delta Ct values were calculated for every gene (Ct_target_—Ct_Gapdh_), and expression levels were calculated as 2^(-ΔCt). Expression levels in microbead-injected versus saline-injected and aged versus young cells were calculated and compared using *t*-tests with Bonferroni correction for multiple comparisons (whole-population comparison). Since expression levels were different for different genes, the data was then normalized for further analysis. Delta Ct values were averaged over all cells in the microbead versus saline and aged versus young groups, respectively. Individual Ct values were subtracted from the averages and divided by the standard deviation. The results were used for generating heatmaps with the rows representing genes and the columns representing individual astrocytes. Then, 95% confidence intervals were calculated for cells from microbead-injected eyes and cells from aged eyes, respectively. If the confidence intervals did not include 0, the genes were considered significantly up- or down-regulated in the microbead (or aged) group.

For morphological analysis of astrocytes, all groups of measurements were first tested for normal distribution (Shapiro–Wilk test), and a non-parametric test (Wilcoxon rank sum test) was used if a group did not pass the test at an alpha level of 0.05. ANOVA with Tukey–Kramer post hoc tests was used to compare IOP measurements and measurements of astrocyte process thickness from electron microphotographs. Linear regression was calculated by the least square method. All values are given as mean ± standard deviation, unless stated otherwise.

### 2.10. Data Sharing

RNA sequencing data was deposited to GEO (GSE139605). Original image stacks of MitoTracker^TM^ Red FM-injected optic nerve heads in B6.*hGFAPpr*::EGFP mice were deposited to the Harvard Dataverse and are accessible under https://dataverse.harvard.edu/dataverse/astrocytes2019 (accessed on 21 August 2023).

## 3. Results

### 3.1. Gene Expression in Optic Nerve Heads from Eyes with and without Elevated IOP

**Figure 1:**
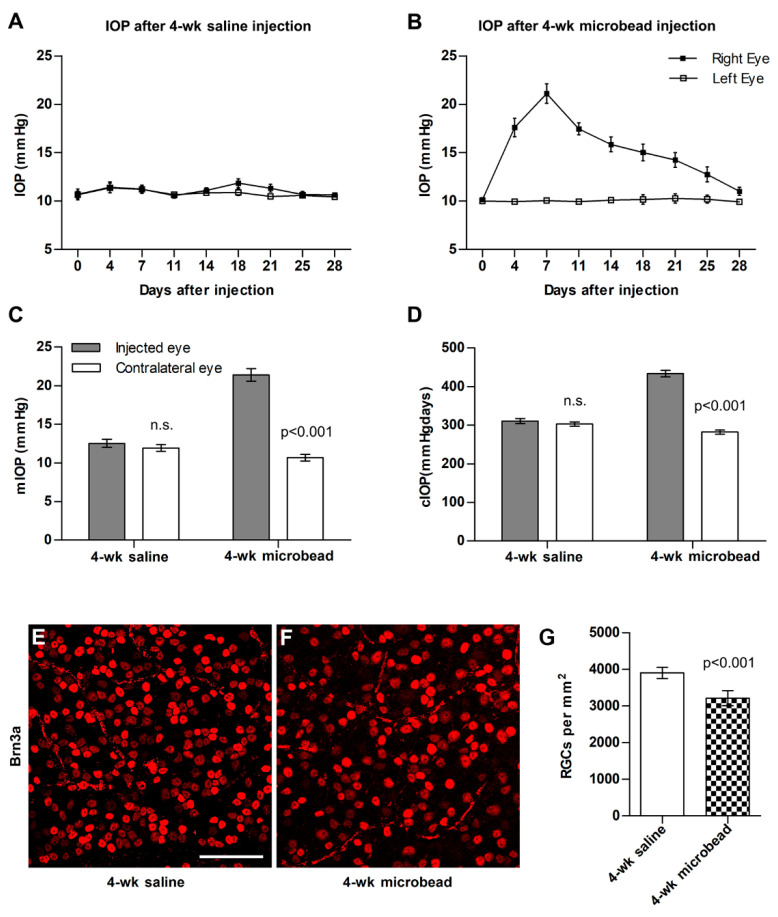
Elevation of IOP and ganglion cell loss in C57bl/6 eyes. (**A**) control animals show no increase in IOP after unilateral injection of sterile saline solution. (**B**) IOP increases after injection of microbeads, peaks at day 7, and gradually returns to baseline. (**C**) Maximum IOP measured during the experimental period in saline- and microbead-injected eyes. (**D**) Cumulative IOP (cIOP, i.e., the area under the curve) of IOP vs. time for saline- and microbead-injected eyes. (**E**) Retinal ganglion cell labeling with Brn3a in a retina 4 weeks after injection of saline solution. (**F**) Retinal ganglion cell labeling with Brn3a in a retina 4 weeks after microbead injection shows marked loss of ganglion cell nuclei. (**G**) 4 weeks of IOP elevation leads to a significant loss of ganglion cells in the injected eyes. Groups were compared using ANOVA with Tukey–Kramer post hoc test. N.s., not significant. Scale bar for panels (**E**,**F**), 25 μm.

**Figure 2:**
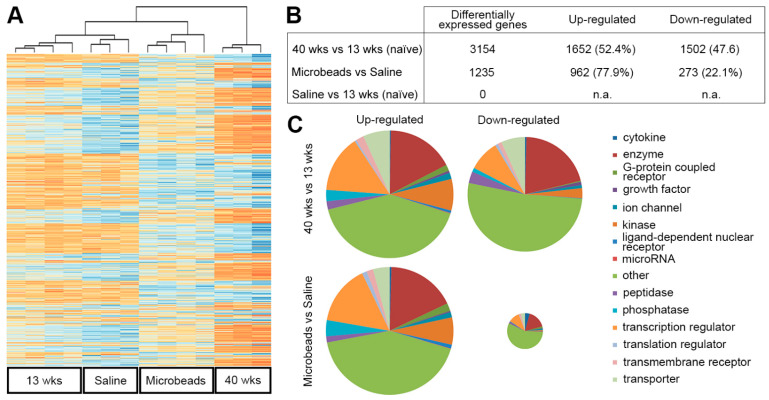
RNA sequencing of optic nerve heads in naïve young, saline-injected (4 weeks after injection), microbead-injected (4 weeks after injection), and naïve aged (40 weeks) mice. (**A**) hierarchical clustering of all differentially expressed genes in the four groups. Differentially expressed genes were defined as genes up- or down-regulated by at least 1.5-fold, *p* < 0.01, FDR = 0.05. Red colors indicate up-regulated genes, blue colors indicate down-regulated genes. The brackets indicate similarities between samples by hierarchical clustering. Saline-injected and young naïve mice cluster together. Microbead-injected and aged nerves form their own clusters, but are overall relatively similar. (**B**) numbers of differentially regulated (DE) genes in microbead (MB) vs. saline-injected nerves and young vs. aged naïve nerves. (**C**) distribution of Ingenuity Pathway Analysis (IPA) annotations for differentially expressed up- or down-regulated genes. The areas of the circles signify the number of total genes in this group. There are roughly the same numbers of up- and down-regulated genes in the aged versus young groups, whereas after microbead injection, about 4 times as many up-regulated genes were detected. n.a., not applicable.

We profiled the gene expression in the glial lamina, the unmyelinated region of the optic nerve (Supplementary Figure S1) of C57bl/6 mice in four groups: a group of young adult mice (13 weeks, *n* = 16, male:female 1:1) that had experienced one month of unilaterally elevated IOP, an age-matched control group (saline injection, *n* = 16), an age-matched (13 weeks) group of naïve mice, and finally a group of aged (10 month) naïve mice. At 13 weeks (young adulthood), the murine retina and optic nerve are fully mature, whereas a 40-week-old mouse would be considered middle-aged [51,52]. This age was chosen because it corresponds to the age when the incidence of glaucoma in humans starts to rise and corresponds roughly to a human age of about 40 years [3]. Injection of microspheres into the right anterior chamber led to an increase in IOP (Figure 1A–D) and a loss of about 15% of ganglion cells in the injected eye one month later (Figure 1E–G). Injection of saline solution did not change IOP or ganglion cell density in the retina (Figure 1A). Total RNA was isolated from 4 optic nerve heads (originating from 2 male and 2 female mice each, Supplementary Table S1) per each of 4 biological replicates. We did not use the contralateral eyes as controls because IOP manipulation in one eye can lead to glial activation in the untreated contralateral eye [53,54]. A total of 14,926 genes had read counts > 10 in our samples. Principal component analysis showed good clustering for all but 2 outliers (one in the Saline group and one in the 40-week naïve group), which were excluded from further analysis (Supplementary Figure S2). We then performed cluster analysis on all groups with the genes that were differentially expressed (*p* < 0.01, FDR = 0.05) in any of the samples, the 13-week-old naïve mice serving as the baseline. As expected, the young naïve and saline-injected groups were very similar, whereas microbead-injected and old naïve nerves formed their own clusters, but showed overall similarity to each other (Figure 2A).

For further analysis of differentially regulated pathways, we directly compared young versus aged naïve and microbead versus saline-injected groups. Differentially expressed genes were defined as genes significantly up- or down-regulated by at least 1.5-fold (2-tailed *t*-test, *p* < 0.01, FDR= 0.05). Figure 2B,C summarize the numbers of differentially expressed genes for these groups and their annotations, respectively (complete gene lists are available under GEO accession number GSE139605). As expected from the overlap of genes differentially expressed in the glaucoma and aged groups, many canonical pathways predicted to be activated or attenuated in glaucoma or age were similar. This included the endothelin-1, interleukin-6, and Stat3 pathways, whose roles in glaucomatous nerves have been described before [12,13,34]. Neither in the glaucoma nor the aged groups did we find up-regulation of cell cycle or cell proliferation-associated pathways.

**Figure 3:**
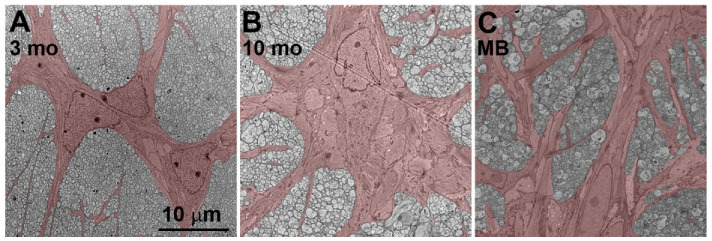
Astrocyte hypertrophy in glaucomatous and aged nerves. (**A**) Transmission electron microphotography of an optic nerve of a 3-month-old naïve C57bl/6 mouse in the coronal plane. The section was taken through the unmyelinated region of the nerve. Astrocyte cell bodies and processes are false-colored in red. (**B**) optic nerve in a 40-week-old naïve mouse. Astrocyte cell bodies and processes are notably enlarged. (**C**) optic nerve in a 3-month-old mouse, 1 month after microbead (MB) injection into the anterior chamber of the eye. Individual processes are 0.97 ± 0.18 µm in 3-month-old nerves, 1.07 ± 0.19 µm in 10-month-old nerves, and 1.16 ± 0.27 µm after microbead injection. Astrocyte processes in aged and glaucomatous mice are significantly different at *p* = 0.0014 and *p* < 0.001 between young and aged, and young and glaucomatous nerves, respectively. ANOVA followed by Tukey-Kramer test.

The similarity between aged and glaucomatous nerves was also apparent in electron microscopy of the unmyelinated optic nerve (Figure 3). Astrocytes in young nerves show the characteristic arrangement of large processes that delineate axon bundles, and small, fine processes that extend into axon bundles (Figure 3A, astrocytes are false-colored in red). Astrocytes in 40-week-old nerves (Figure 3B) or nerves after one month of IOP elevation (Figure 3C) show obvious process hypertrophy. We did not observe more astrocyte nuclei, as an indicator of cell numbers, in old or glaucomatous nerves (4 glaucomatous nerve profiles and 5 age-matched normal nerve profiles were counted).

### 3.2. Differences between Microbead-Injected and Aged Nerves

We compared genes differentially expressed in glaucomatous versus aged nerves to identify biological functions that are activated specifically in optic nerves after 4 weeks of elevated IOP (Table 1). Several of the pathways predicted to be activated in glaucomatous versus aged nerves are annotated with the formation of protrusions, sprouting, and branching of cells. We therefore asked whether the functional consequence of newly formed astrocyte processes may be an increase in phagocytic activity.

### 3.3. Expression of Phagocytic Markers in Individual Astrocytes

Astrocytes are the major cell type in the optic nerve, but microglia, endothelial cells, and fibroblasts from the surrounding pia are also present, and some of the gene expression changes in our study may be due to contributions from these cell types. To validate astrocyte-specific gene expression, we therefore isolated single astrocytes from freshly dissociated optic nerves 2 weeks after microbead injection (high IOP) or saline injection (controls). Aged astrocytes were collected from 40-week-old naïve animals; astrocytes from young naïve nerves were collected as controls. Astrocytes retain their morphology well after dissociation [48], but in every case the cell type was also confirmed by verifying that the cells were positive for the astrocyte marker GFAP and negative for the microglial marker Iba1 and the oligodendrocyte marker MBP by conventional PCR. Phagocytosis-related genes (*Abca1*, *Abca7*, *Dock1*, *Gulp1*, *Lamp1*, *Lgals3*, *Megf10*, *Mfge8*), genes related to the degradation of mitochondria (*Pink1*, *Park2*), and markers of astrocyte reactivity (*Fbln5*, *H2-T23*, *Psmb8*, *Srgn*, A1 reactivity; *Clcf1*, *Emp1*, *Ptx3*, *Stat3*, *Tgm1*, A2 reactivity) were tested using quantitative PCR.

**Figure 4:**
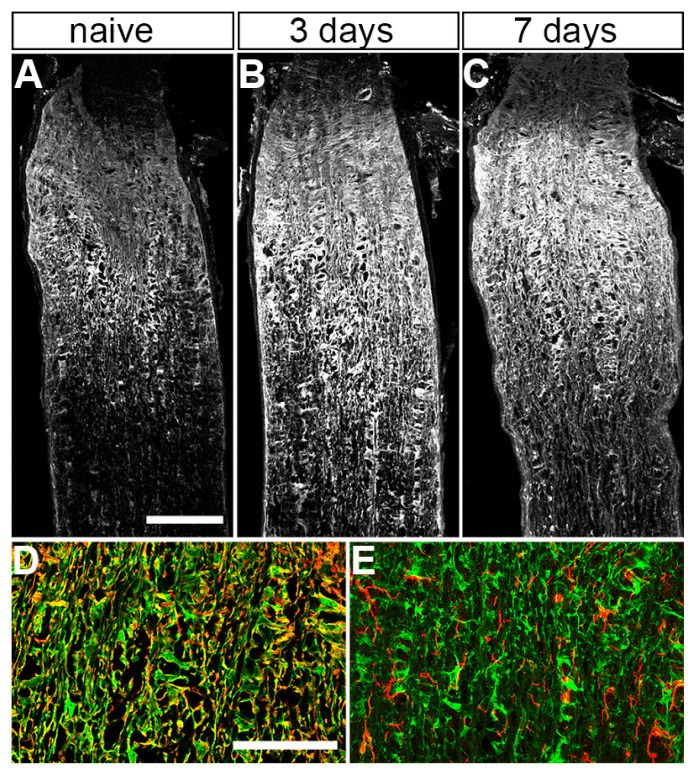
Apparent increase in staining for Galectin-3 after IOP elevation. (**A**) longitudinal section of a naïve optic nerve immunolabeled for Galectin-3. (**B**,**C**) 3 and 7 days after microbead injection. Galectin-3 staining is observed in the naïve control, but qualitatively appears to be up-regulated at 3 and 7 days. Scale bar for A-C, 100 μm. (**D**) higher magnification of an optic nerve 7 days after microbead injection stained for Galectin-3 (green) and the astrocyte marker GFAP (red). (**E**) higher magnification of a nerve 7 days after microbead injection stained for Galectin-3 and the microglia marker IBA1 (red). Scale bar for (**D**,**E**), 50 μm.

**Figure 5:**
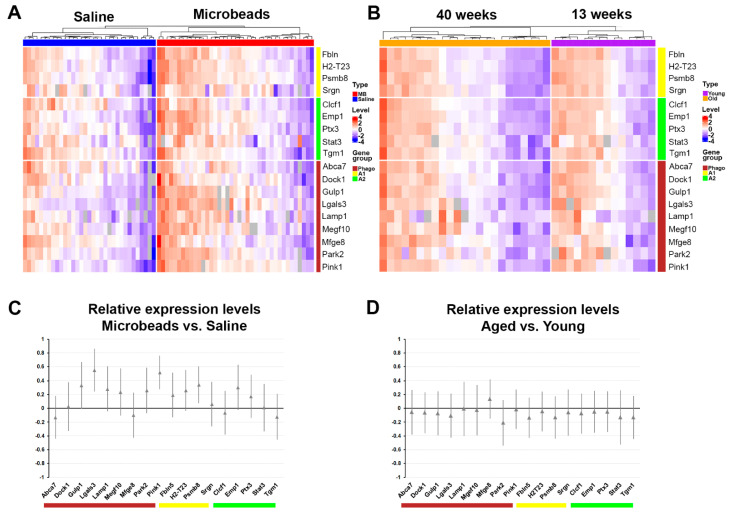
Single-cell qPCR of saline- vs. microbead-injected and young vs. aged astrocytes. (**A**) heatmap of normalized expression values in single, freshly dissociated astrocytes after microbead- or saline-injection. Rows represent genes (A1 reactivity markers (yellow bar) *Fbln5*, *H2-T23*, *Psmb8*, *Srgn*; A2 reactivity markers (green bar) *Clcf1*, *Emp1*, *Ptx3*, *Stat3*, *Tgm1*; phagocytosis-related markers (dark red bar) *Abca7*, *Dock1*, *Gulp1*, *Lgals3*, *Lamp1*, *Megf10*, *Mfge8*; mitochondrial degeneration markers *Park2*, *Pink1*). Columns represent individual astrocytes (*n* = 33 for the saline group (blue bar), *n* = 39 for the microbead group (red bar)). (**B**) heatmap of normalized expression values in single astrocytes from naïve 13-week (purple bar) or 40-week (orange bar) old C57bl/6 mice. Genes are the same as for A. Columns represent individual astrocytes (*n* = 14 for the 3-month group, *n* = 23 for the 9-month group). (**C**) Normalized expression levels for all cells from microbead-injected nerves compared to the population average. A value of 0 would indicate that the distribution of high-expressing and low-expressing cells are identical for the microbead-injected and control (saline) group. A value of 1 would indicate that all microbead-injected cells are in the high expressing group. For most genes, more astrocytes in the microbead-injected group are high-expressing; however, this reached significance (defined as the 95% confidence interval for the gene not containing 0) only for *Gulp1*, *Lgals3*, *Pink1*, and *Psmb8*. Grey bars represent 95% confidence intervals. (**D**) Same analysis as in C for 13-week and 40-week-old naïve astrocytes. No gene is expressed at a significantly higher or lower level in the older group (all confidence intervals include 0).

Considering the whole population of astrocytes in our sample, *Gulp1* and *Lgals3* were significantly up-regulated in astrocytes from microbead- versus saline-injected nerves (*Gulp1*, 2.5-fold, *p* = 0.00056 and *Lgals3*, 7.8-fold 7.2 × 10^−5^, *t*-test with Bonferroni correction for multiple comparisons). This was also apparent in immunostaining for the gene product of *Lgals3*, galectin-3, which is up-regulated after microbead injection (Figure 4A–C). Galectin-3 co-localized with the astrocyte marker GFAP, but not with the microglia marker IBA1 (Figure 4D,E). Amplification of *Abca1* was weak or below the detection limit for most astrocytes, though *Abca1* is present in RNA samples from the whole optic nerve, suggesting that it may localize mainly to cells other than astrocytes. Most of the overall activation of engulfment and phagocytosis genes is driven by a subset of highly active cells, rather than by a shift of the whole population to a more phagocytic state (Figure 5A). There was no clear trend towards an A1 (neurotoxic) or A2 (protective) type of astrocyte reactivity for the genes tested after microbead injection (Figure 5A,C). In contrast, neither phagocytosis-related genes nor markers of reactivity were up-regulated in aged versus young naïve astrocytes (Figure 5B,D).

### 3.4. Mitochondria Incorporation into Optic Nerve Head Astrocytes

**Figure 6:**
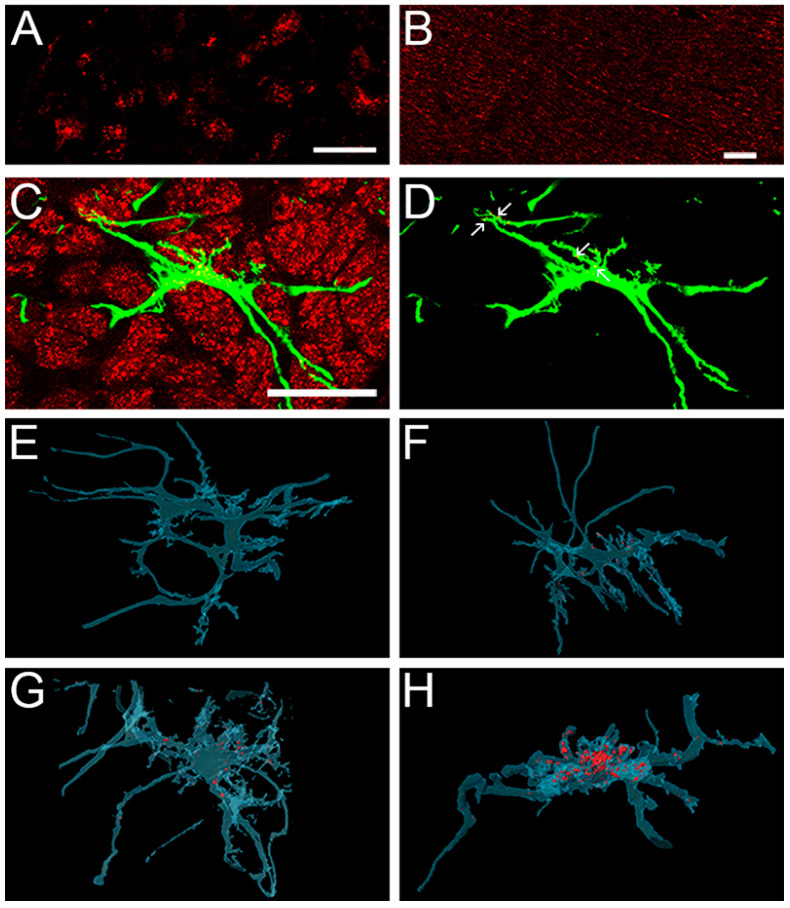
Distribution of axonal mitochondria in surrounding astrocytes. MitoTracker^TM^ Red FM labeling. (**A**) Whole mounted retina 4 days after injection of MitoTracker^TM^ Red FM into the contralateral superior colliculus. Retrograde axonal transported labeled mitochondria appear as puncta in the retina. Scale bar, 20 µm. (**B**) Longitudinal section of the optic nerve 4 days after injection of MitoTracker^TM^ Red FM. Scale bar, 20 μm. (**C**) Cross section of an optic nerve (unmyelinated region) with a single astrocyte expressing EGFP. Axonal mitochondria were labeled by injection of MitoTracker^TM^ Red FM into the contralateral superior colliculus. The majority of mitochondria does not co-localize with the astrocyte. Scale bar, 50 μm. (**D**) Same astrocyte after digitally removing all mitochondria signals that were not enclosed within EGFP^+^ astrocyte cytoplasm in all 3 dimensions. Several labeled mitochondria (arrows in (**D**)) are visible inside the astrocyte. (**E**–**H)** representative images of astrocytes with no incorporated mitochondria. (**E**), sporadic (**F**), moderate (**G**) and large amounts of mitochondria incorporation. The images were reconstructed using the Amira software package after digitally removing the mitochondria not incorporated into an EGFP^+^ astrocyte. The astrocyte is rendered transparent to show the mitochondria inside.

Astrocytes in the unmyelinated segment of the optic nerve are known to incorporate axonal mitochondria [55]. We made use of this property to directly visualize phagocytic activity in optic nerve head astrocytes in vivo. A cohort of B6.*hGFAPpr*::EGFP mice (10 animals) were unilaterally injected with microspheres solution into the anterior chamber (Supplementary Figure S3). In this strain, EGFP is expressed in astrocytes in a stochastic manner so that individual cells can directly be observed microscopically [8,18]. In addition, each animal received an injection of MitoTracker^TM^ Red FM into the contralateral superior colliculus on day 13 after microbead injection. Another group of 10 B6.*hGFAPpr*::GFP mice received the MitoTracker^TM^ Red FM injection without prior injection of microbeads. Four days later (day 17) the mice were sacrificed. These timepoints were chosen because the IOP elevation is at a relatively stable plateau between days 11 and 21 (Figure 1B). MitoTracker^TM^ Red FM is taken up by the ganglion cell axonal terminals and labels mitochondria that are transported retrogradely to the cells’ somata in the retina. Since there is no other direct connection, all labeled mitochondria in the optic nerve must be axonal in origin. Labeled mitochondria appeared in the retinal ganglion cell bodies (Figure 6A) and axons in the optic nerve (Figure 6B) four days later in the high-IOP and control groups. We tested whether any MitoTracker^TM^ Red FM-labeled axonal mitochondria become incorporated by astrocytes by confocal imaging of EGFP^+^ cells. A total of 38 image stacks containing 344 unique EGFP^+^ astrocytes were imaged in the control group, and 34 image stacks containing 339 astrocytes were imaged in the high-IOP group.

**Figure 7:**
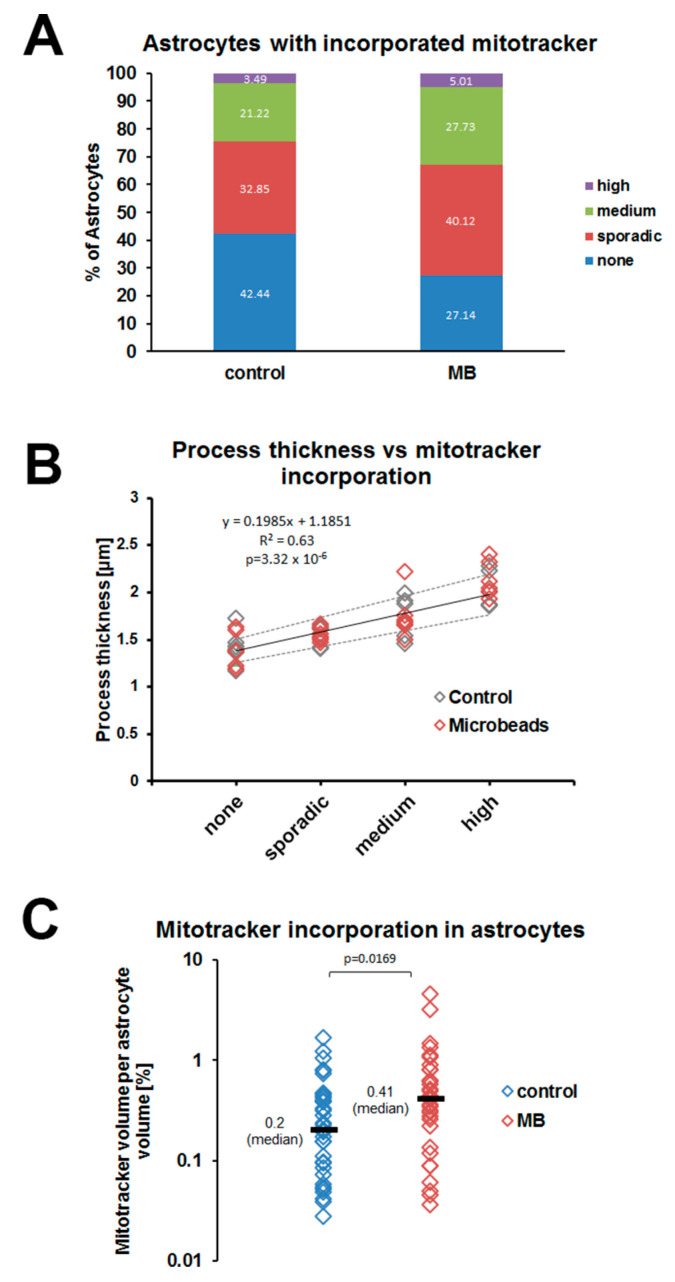
Quantification of mitochondria incorporation into optic nerve astrocytes. (**A**) percentages of astrocytes with no, sporadic, medium, and high incorporation of axonal mitochondria encountered in saline-injected and microbead-injected nerves. (**B**) Process thickness of astrocytes with no, sporadic, medium, and high incorporation of MitoTracker^TM^ Red FM. Process thickness was measured 10 μm distal to the soma in 6 astrocytes from each category in saline-injected (control, grey diamonds) and microbead-injected (red diamonds) nerves; in total *n* = 24 astrocytes in each group. Solid grey line shows the linear regression line based on the control cells. Dashed grey lines show 95% confidence intervals. There was no significant difference between astrocytes from control and injected nerves. (**C**) total MitoTracker^TM^ Red FM incorporation in astrocytes (in % of the total EGFP+ astrocyte volume) in saline-injected and microbead-injected (MB) nerves. Diamonds indicate individual cell measurement, Wilcoxon rank sum test.

As most of the MitoTracker^TM^ Red FM-labeled mitochondria do not co-localize with EGFP^+^ astrocytes (Figure 6C), we digitally reconstructed each image stack including only those mitochondria that were surrounded in all three dimensions by astrocytic cytoplasm (Figure 6D). Not all astrocytes incorporated labeled mitochondria (Figure 6E–H). In the control group 146 out of 344 astrocytes contained no MitoTracker^TM^ Red FM (42.4%). This number was lower in the high IOP group (92 out of 339; 27.1%) (Figure 7A). The amount of incorporated MitoTracker^TM^ Red FM per astrocyte differed considerably between individual cells within the same nerve. Astrocytes located between 0–100 μm behind the sclera contained few labeled mitochondria. In addition, incorporated mitochondria were not distributed evenly within individual astrocytes. Most labeled material was observed either in the cell body or in short, tortuous, or longitudinal processes. The long, relatively smooth primary astrocyte processes contained relatively few MitoTracker^TM^ Red FM-labeled mitochondria. Astrocytes that contained labeled mitochondria generally also showed process hypertrophy (Figure 7B). This was the case for astrocytes from both saline-injected nerves and microbead-injected nerves.

To quantify whether an increase in IOP leads to increased incorporation of mitochondria, we calculated the volume of MitoTracker^TM^ Red FM-labeled mitochondria in astrocytes for every image stack in the control and high-IOP group. Since EGFP expression in the B6.*hGFAPpr*::GFP strain is stochastic, the number of labeled astrocytes is different from nerve to nerve. Therefore, we also determined the total volume of EGFP^+^ astrocytes per stack and calculated the incorporation of labeled mitochondria per astrocyte volume (Figure 7C). The fraction of astrocyte volume occupied by MitoTracker^TM^ Red FM-labeled material was generally small (range 0–1.63% of the volume, median 0.2% for the control group). In the high IOP group, this amount was moderately increased (range 0.036–4.52%, median 0.41%, *p* = 0.0151, Wilcoxon rank sum test).

## 4. Discussion

### 4.1. Astrocytes from Aged and Glaucomatous Optic Nerve Heads Appear Similar in Many Respects

Aging has long been recognized as a risk factor for glaucoma and confounding factor in diagnosis since glaucomatous eyes usually are older eyes [56,57]. Age-related changes of course occur in all ocular tissues [58], and though any single one of them may not be clinically relevant by itself, they can jointly contribute to the severity of glaucoma [59]. Most importantly in this context, in all mammalian species where this question has been addressed, ganglion cell axon counts were found to decrease with age [60,61]. Though the rate of axon loss dramatically increases in the presence of elevated IOP [62], similar changes in astrocyte reactions and axon numbers may be expected in glaucomatous and aged optic nerves, albeit on different time scales. This is indeed observed for optic nerve axons and blood vessels, so that glaucomatous nerves appear prematurely aged [35]. Not unexpectedly, the morphological similarity of aged and glaucomatous nerves corresponds to a similar pattern of gene expression changes in both conditions. RNA extracted from the unmyelinated segment of the optic nerve mainly originates from astrocytes, which are the major cell type of this region, whereas microglia and endothelial cells are sparse [6,8]. RNA-seq experiments showed an overall similarity of gene expression profiles in aged and glaucomatous astrocytes (Figure 2A), and especially for up-regulated genes the overlap between both sets was high (77.9% of genes up-regulated after IOP elevation are also up-regulated in aged nerves). We did not find an enrichment of genes associated with cell cycle or proliferation, and counts of astrocyte nuclei in electron microscopy images showed no difference between nerves after one month of IOP elevation and controls. This differs from earlier reports that described astrocyte proliferation in glaucomatous nerves of DBA/2J mice or rats with experimentally induced ocular hypertension [13,63,64,65]. A possible explanation is that the microbead model used in our study models the early response to a moderate increase in IOP that is not severe or long-lasting enough to induce astrocyte proliferation. However, both in aging and glaucomatous nerves astrocyte processes showed hypertrophy, a sign of a more reactive phenotype [66,67,68]. Most genes associated with the A1 (neurotoxic) or A2 (neuroprotective) route of astrocyte reactivity were not differentially expressed either in the microbead or the aged groups. This was surprising as a recent study demonstrated elevation of A1 genes in murine optic nerves after IOP elevation [69]. A possible explanation for this discrepancy may be the use of different platforms (microfluidic qPCR versus RNA-sequencing).

### 4.2. Sprouting of New Astrocytic Processes Is an Early Event in Glaucoma

The main difference between aged and glaucomatous nerve heads was in the up-regulation of pathways annotated with cell sprouting and the formation of processes. This agrees with previous morphological studies in the optic nerves of glaucomatous DBA/2J mice and C57bl/6 mice after one month of IOP elevation [14,18]. Astrocytes in the glial lamina are normally fairly flat and oriented in the coronal plane—i.e., their processes span the diameter of the optic nerve but have only very few and short longitudinal processes [8]. In glaucomatous nerves, the astrocytes extend new processes that run longitudinally in the optic nerve, invade axon bundles, and often contain remnants of mitochondria. However, it was not clear what cell biological function these new processes carried out. Astrocytes are known to be phagocytic in vitro and in vivo [31,70,71,72,73,74]. A particularly intriguing mechanism links the astrocytes of the optic nerve to the degradation of axonal mitochondria. In a process described as “transcellular mitophagy”, astrocyte processes enwrap axonal evulsions that contain degenerating mitochondria, and eventually internalize them [55,75]. This process is physiological and presumably helps retinal ganglion cells to dispose of mitochondria that have reached the end of their lifespan, but there is also evidence that axonal evulsions are particularly numerous in the optic nerves of glaucomatous DBA/2J mice [76].

We therefore measured the incorporation of axonal mitochondria into astrocytes in a double-label in vivo assay. Interestingly, both in naïve and glaucomatous nerves, we observed large differences in the amount of material any individual astrocyte incorporated. In all nerves, there were at least some cells that did not contain any labeled material, whereas a small number of cells were highly active. Glaucomatous nerves contained more highly active cells, but even in those nerves some astrocytes remained that had incorporated very little or no axonal mitochondria. Cells that had incorporated many labeled mitochondria also showed hypertrophy of their processes, a finding commonly associated with a more reactive state (Figure 7B). The heterogeneity of astrocytes in normal and glaucomatous nerves was also apparent in single-cell qPCR for phagocytosis- and reactivity-associated genes. Though on the population level the expression of *Lgals3* and *Gulp1* was increased after elevation of IOP, the increase was mainly due to an increase in numbers of highly active cells rather than a shift of the whole population.

### 4.3. Implications of the Apparent Heterogeneity of Optic Nerve Head Astrocytes

Taken together, our results suggest that at any time a subset of optic nerve head astrocytes is reactive—as indicated by process hypertrophy, high phagocytic activity, and relatively high expression of reactivity genes (however without a clear bias towards the A1 or A2 phenotype described for protoplasmic astrocytes in other regions of the brain). An episode of IOP elevation leads to an increase of astrocyte reactivity on the population level. However, we did not find that the astrocytes move in unison towards a more reactive phenotype. Neither were reactive astrocytes in glaucomatous nerves obviously different from their counterparts in naïve nerves—actually, they were indistinguishable by all parameters we tested. The only difference was that the number of reactive astrocytes was higher in the glaucomatous nerve heads. For example, the percentage of astrocytes that had not incorporated any axonal mitochondria was 42.44% in control nerves and decreased to 27.14% in nerves after IOP elevation (Figure 7).

There are several possible interpretations of this observation. Perhaps the easiest is that there are (at least) two different types of astrocyte present in the optic nerve head, only one of which is highly phagocytic and prone to becoming reactive. However, there is no clear evidence on the anatomical level that the astrocytes of the glial lamina belong to different types. Rather, their shape is fairly uniform [8,77]; and without the clear morphological distinctions that usually form the basis of type definitions in other systems [78]. In addition, all astrocytes in our single cell PCR expressed most (usually all) phagocytic and reactivity markers tested, though not necessarily at the same levels. This suggests that differences between individual astrocytes in the optic nerve head are a matter of degree, not of clearly identifiable types.

Another explanation would be that astrocytes react to local signals in their vicinity, such as “find me” signals like ATP or UDP leaking out of injured axons, or microglia-derived factors [49,79]. Such signals might instruct an astrocyte to extend new processes towards the origin of the signal or increase its phagocytic activity. Yet, the anatomy of the optic nerve head argues against this explanation. In contrast to protoplasmic astrocytes in the grey matter of the CNS, which occupy spatially separated domains [80,81], the astrocytes in the optic nerve head often span the whole diameter of the nerve and their processes overlap considerably forming the sieve-like meshwork of the glial lamina [8]. It is hard to imagine how a localized signal would affect only a subset of the closely intermingled astrocyte processes.

An alternative interpretation of our observations would be that astrocytes cycle through periods of high phagocytic activity followed by a period of relative inactivity. Astrocytes are not professional phagocytes and are relatively slow in degrading and eliminating phagocytized material [82], which may explain why a resting period is needed. Since it is at present not possible to follow individual optic nerve astrocytes in vivo in the intact optic nerve, our experiments necessarily show only a static snapshot of what is most likely a continuous physiological process. Cells that were not active during the period the MitoTracker^TM^ Red FM was present would have remained unlabeled, and hence invisible to our observations. The implication would be that optic nerve head astrocytes are always at least somewhat reactive. This may be caused by the constant mechanical strain they are exposed to as the IOP fluctuates due to circadian rhythm, eye movements, and posture even in healthy individuals [83,84,85]. Astrocytes express several mechanosensitive channels and extend long processes that insert into the pial sheet around the nerve [8,48,86,87,88,89]. They are therefore in an ideal anatomical position to sense stretch on the optic nerve head imparted through IOP fluctuations. In the presence of elevated IOP, more astrocytes might transition to the reactive state, or alternatively, activated cells are prevented from returning to the resting level.

## 5. Conclusions

Life cell imaging and gene expression surveys have expanded our understanding of astrocytes and their reaction to injury. Experimental evidence indicates that reactive astrocytes perform many functions that are beneficial for neuronal survival, especially in the early stages of glaucoma. Hopefully, stimulating the beneficial (and inhibiting the harmful) activities of reactive astrocytes can be used as a neuroprotective approach to glaucoma therapy.

## Figures and Tables

**Table 1:** Ingenuity Pathway Analysis annotation of the major predicted up- and down-regulated pathways in microbead-injected nerves versus aged nerves.

Annotation	*p* Value	Direction	z Score
Formation of cellular protrusions	1.98 × 10^−9^	Increased	2.705
Development of neurons	1.01 × 10^−8^	Increased	2.441
Infection of cells	1.08 × 10^−6^	Increased	3.768
Sprouting	4.63 × 10^−6^	Increased	2.484
Cell movement of tumor cell lines	1.31 × 10^−5^	Increased	2.037
Branching of neurites	4.19 × 10^−5^	Increased	2.991
Cytostasis	0.000154	Increased	2.411
Inflammatory response	0.000213	Increased	3.093
Cell movement of phagocytes	0.000292	Increased	2.112
Nonhematologic malignant neoplasm	3.18 × 10^−33^	Decreased	−2.45
Development of genitourinary system	4.66 × 10^−7^	Decreased	−2.435
Neurodegeneration of cerebellum	5.49 × 10^−5^	Decreased	−2.8
Neurodegeneration of Purkinje cells	8.68 × 10^−5^	Decreased	−2.621
Abnormality of heart ventricle	0.000299	Decreased	−2.126

## Data Availability

Confocal image stacks are available at https://dataverse.harvard.edu/dataverse/astrocytes2019. RNA-sequencing data has been submitted to GEO (Accession number GSE139605).

## Supplementary Materials

The following supporting information can be downloaded at: https://www.mdpi.com/article/10.3390/cells12172131/s1, Table S1: Group Summaries for RNA-sequencing; Table S2: Primer Sequences; Figure S1: Schematic of the Optic Nerve; Figure S2: Principal Component Analysis; Figure S3: Intraocular Pressure.
