# Supplementary material for: Astrocytes in the Optic Nerve Are Heterogeneous in Their Reactivity to Glaucomatous Injury
Source: Cells. 2023 Aug 23;12(17):2131. doi: 10.3390/cells12172131 (PMC10486930; doi:10.3390/cells12172131)
Supplement: Supplementary file 1 [file cells-12-02131-s001.zip › cells-2165527-supplementary.pdf]

Supplementary Materials

Supplementary Table S1: Group Summaries for RNA Sequencing

Group 1	13-week-young naïve			
Samples	Sample Number	RIN	Concentration	
1	YNR1	9.2	2298	pg/µl
2	YNR2	9.2	3469	pg/µl
3	YNR3	9.7	3859	pg/µl
4	YNR4	9.8	2498	pg/µl

Group 2	40-week-old naïve			
Samples	Sample Number	RIN	Concentration	
1	ONR1	9.5	26187	pg/µl
2	ONR2	9.8	11981	pg/µl
3	ONR3	9.7	14769	pg/µl
4	ONR4	9.9	12499	pg/µl

Group 3	4-week saline injection group			
Samples	Sample Number	RIN	Concentration	
1	4SR1	9.2	1170	pg/µl
2	4SR2	9.5	600	pg/µl
3	4SR3	9.3	543	pg/µl
4	4SR4	9.3	3115	pg/µl

Group 4	4-week bead injection group			
Samples	Sample Number	RIN	Concentration	
1	4BR1	9.1	2363	pg/µl
2	4BR2	9	4175	pg/µl
3	4BR3	9.2	3718	pg/µl
4	4BR4	9.3	3380	pg/µl

Supplementary Table S2: Primer sequences

	Forward	Reverse
**Abca1**	5’-gcttgttggcctcagttaagg-3’	5’-gtagctcaggcgtacagagat-3’
**Abca7**	5’-aattacacctatcgacggagaca-3’	5’-tgacggacagccactagga-3’
**Clcf1**	5’-gactcgtgggggatgttagc-3’	5’-ctaagctgcggagttgatgct-3’
**Dock1**	5’-caggaagcataaatacctcgcc-3’	5’-cagctcatccgattgtctttgt-3’
**Emp1**	5’-ttggtgctactggctggtct-3’	5’-cattgccgtaggacagggag-3’
**Fbln5**	5’-gcttgtcgtggggacatgat-3’	5’-tggggtagttggaagctggta-3’
**Gapdh**	5’-ggttgtctcctgcgacttcaa-3’	5’-cctgttgctgtagccgtattcat-3’
**Gfap**	5’- cacgaacgagtccctagagc-3’	5’-atggtgatgcggttttcttc-3’
**Gulp1**	5’-acagaagttgtgagagatgctg-3’	5’-gcagttgtgttgaacctccttt-3’
**H2-T23**	5’-acagtcccgacccagagtag-3’	5’-ccacgtagccgacaatgatga-3’
**Lamp1**	5’-cagcactctttgaggtgaaaaac-3’	5’-acgatctgagaaccattcgca-3’
**Lgals3**	5’-ggagagggaatgatgttgcct-3’	5’-tcctgcttcgtgttacacaca-3’
**Megf10**	5’-gaagaccccaacgtatgcag-3’	5’-cggtgcagcttgtgtagtaga-3’
**Mfge8**	5’-agatgcgggtatcaggtgtga-3’	5’-ggggctcagaacatccgtg-3’
**Oaz1**	5’-ccactgcttcgccagagag-3’	5’-ccccggacccaggttacta-3’
**Park2**	5’-tcttccagtgtaaccaccgtc-3’	5’-ggcagggagtagccaagtt-3’
**Pink1**	5’-ttcttccgccagtcggtag-3’	5’-ctgcttctcctcgatcagcc-3’
**Psmb8**	5’-atggcgttactggatctgtgc-3’	5’-cgcggagaaactgtagtgtcc-3’
**Ptx3**	5’-cctgcgatcctgctttgtg-3’	5’-ggtgggatgaagtccattgtc-3’
**Srgn**	5’-ctcgccttcgtcctggttt-3’	5’-cctcgatgcagttcgcaaaaa-3’
**Stat3**	5’-caataccattgacctgccgat-3’	5’-gagcgactcaaactgccct-3’
**Tgm1**	5’-tctgggctcgttgttgtgg-3’	5’-aaccagcattccctctcgga-3’

Supplementary Figure S1: Schematic of the optic nerve

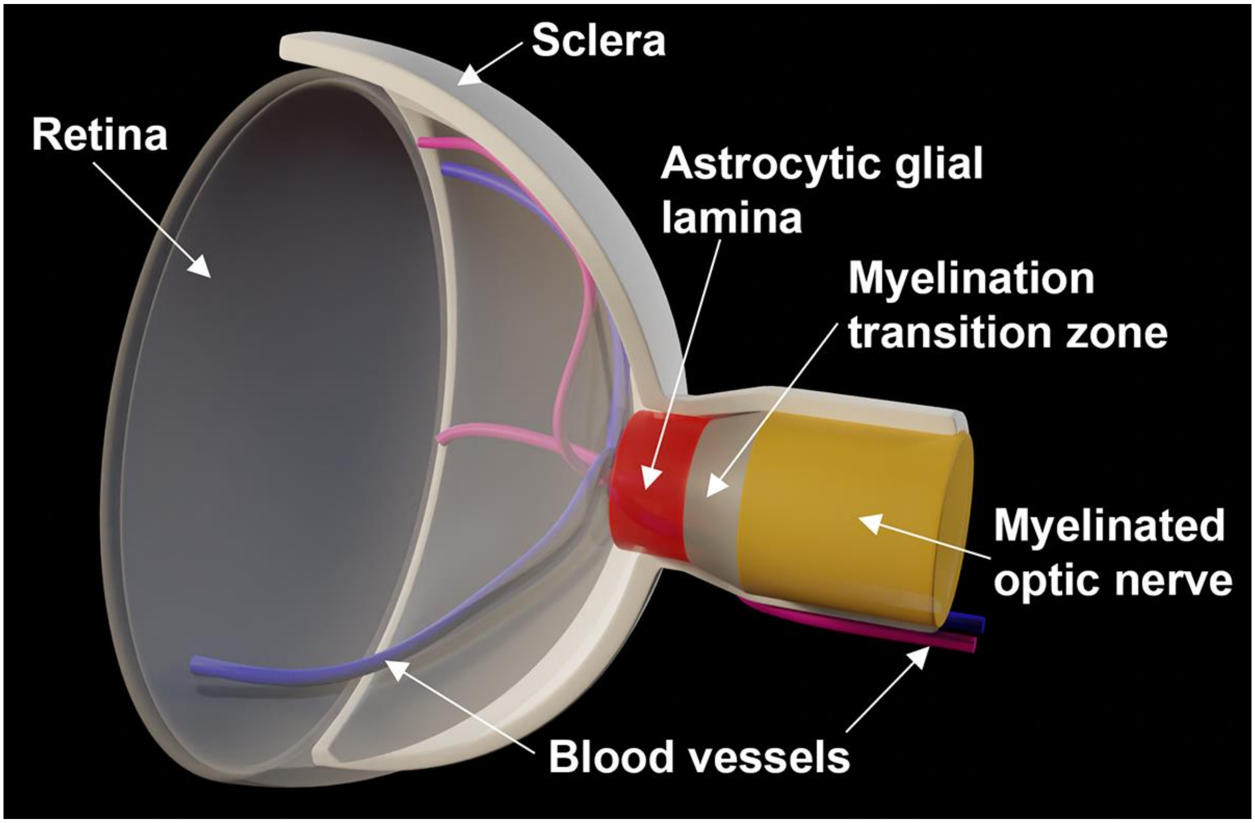

Supplementary Figure S2: Principal Component Analysis

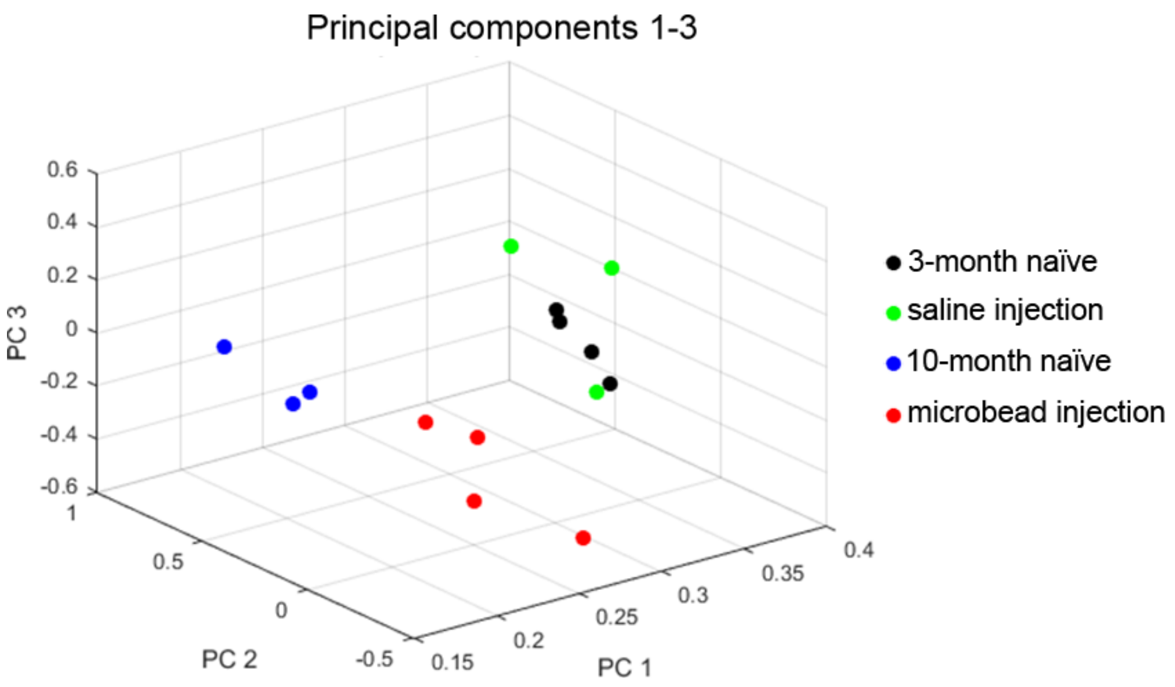

Scatter plot of the first 3 components, Principal Component Analysis of differentially expressed genes in microbead-injected nerves (red symbols), saline-injected nerves (green), young naïve nerves (black), and aged naïve nerves (blue).

Supplementary Figure S3: Intraocular pressure (IOP) after microbead injection

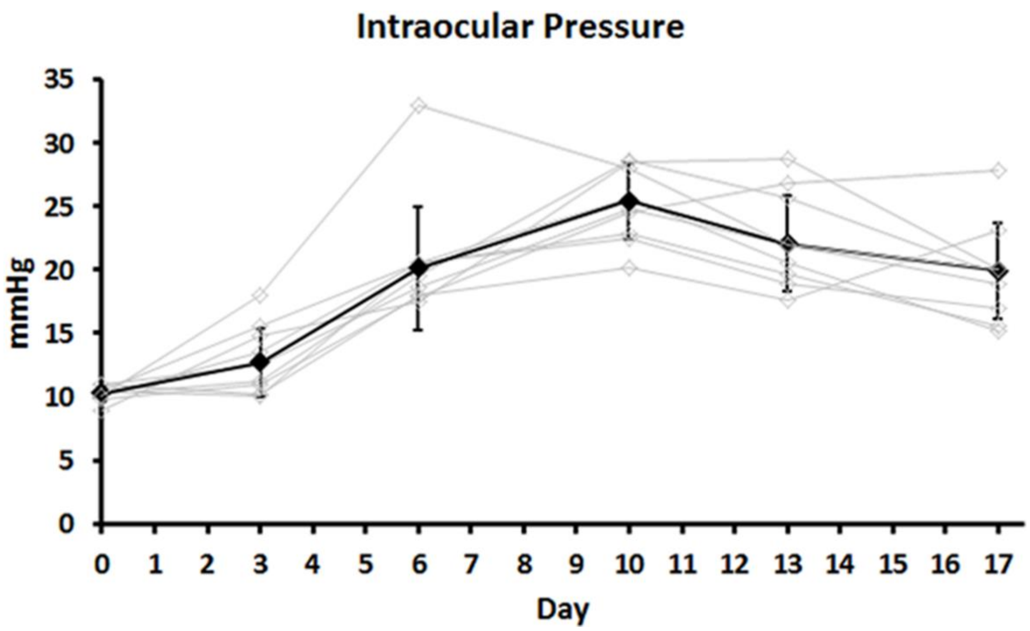

IOP curves after microbead injection into the anterior chambers of B6.*hGFAPpr*::EGFP mice. Thin grey lines represent individual eyes, and the bold black line shows the population average. Error bars represent the standard deviation.
